# Nitrate supply to grapevine rootstocks – new genome-wide findings

**DOI:** 10.1093/jxb/erx273

**Published:** 2017-09-05

**Authors:** Anna Medici, Benoit Lacombe, Sandrine Ruffel

**Affiliations:** Laboratoire de Biochimie et Physiologie Moléculaire des Plantes, UMR CNRS/INRA/SupAgro/UM, Institut de Biologie Intégrative des Plantes ‘Claude Grignon’, Place Pierre Viala, Montpellier, France

**Keywords:** Grafting, grapevine, nitrate, RNA-seq, rootstocks, scion–rootstock couple, transcriptome, viticulture

## Abstract

This article comments on:

Cochetel N, Escudié F, Cookson SJ, Dai Z, Vivin P, Bert P-F, Muñoz MS, Delrot S, Klopp C, Ollat N, Lauvergeat V. 2017. Root transcriptomic responses of grafted grapevine to heterogeneous N availability depend on rootstock genotype. Journal of Experimental Botany **68**, 4339–4355.

**Understanding the plant response to nitrate availability is crucial for sustainable agriculture. In viticulture, there is an additional element to consider: the choice of scion–rootstock couple, which allows the management of environmental cues (including nitrate availability) and productivity. Using the two rootstocks 1103 Paulsen and Riparia Gloire de Montpellier, known to confer different vigour to grafted Cabernet Sauvignon scions, Cochetel *et al.* (2017) have now performed the first genome-wide transcriptome study indicating the genetic basis of the response to heterogeneous nitrate supply in this situation.**

Some of the first written evidence of the use of grafting for grapevine (*Vitis vinifera*) cultivation is an ancient Sumeric text, interpreted by historians as referring to a technique for coping with an environmental stress (Mudge *et al.*, 2009). From its ancient discovery through development in the 19th century, today grafting is applied to more than 70 woody perennial crop species cultivated for their fruits (Warschefsky *et al.*, 2016). As reported by Warschefsky *et al.* (2016), among the wide list of primary targets for rootstock selection is a capacity to confer tolerance to biotic and abiotic stresses (such as nutrient scarcity), and in parallel to assure the best possible productivity of the scion. Nitrogen availability and nitrogen-use efficiency (NUE) are now high-priority research topics in the context of sustainable agricultural development (Zhang *et al.*, 2015) and, during recent decades, the molecular mechanisms of nitrogen sensing in fluctuating environments have been deciphered (reviewed by O’Brien *et al.*, 2016).

Different rootstocks can differently modulate the growth of a given grapevine scion. For example, a Cabernet Sauvignon (CS) scion grafted onto a Riparia Gloire de Montpellier (CS/RGM) rootstock is less vigorous than the same CS scion grafted onto a 1103 Paulsen (1103P) rootstock (Lecourt *et al.*, 2015). The Cochetel *et al.* paper is the first report on the transcriptional reprogramming which occurs in these rootstocks (RGM and 1103P) when subjected to a nitrate-varying environment. The experimental set-up allowed the identification of modules of genes that were: (i) specifically linked to a rootstock genotype, (ii) common to the grapevine root response to nitrate and (iii) specific to the nitrate response of one variety of rootstock. The large amount of information obtained in this work on rootstock-specific gene modules will be a valuable source of molecular markers for selecting top-performing rootstock–scion combinations.

## Hubs for nitrate-responsiveness of rootstocks

Studying gene regulatory networks allows the identification of hubs, which are potentially key regulators of a specific condition (genotype and/or treatment). This approach was used to identify key factors that regulate nitrogen-responsive networks in Arabidopsis (Gutiérrez *et al.*, 2008; Canales *et al.*, 2014). Even though phylogenetically distant, Cochetel *et al.* have now demonstrated that grapevine and Arabidopsis share a common set of genes regulated by nitrate availability. For example, they show that VvNRT2.4a, a member of the high-affinity nitrate transporter family, is modulated by N supply in the two rootstocks.

Searching for N-specific/rootstock-specific hubs, they found two candidates: a BTB-like protein-coding gene and a TCP20 homologue. The BTB-like coding gene belongs to the modules correlating with high nitrate supply in RGM. In Arabidopsis and rice BT2, a BTB-like protein, is involved in the repression of high-affinity nitrate transporters in low nitrate conditions (Araus *et al.*, 2016). In RGM roots, Cochetel *et al.* found higher VvNRT2.4a expression levels and nitrate content. In spite of this difference between the two species, BTB proteins were found to modulate nitrogen-use efficiency and to have an impact on leaf development (Araus *et al.*, 2016), suggesting a role of BTB-like proteins in the control of CS scion growth by RGM. In parallel to this, the second hub found by Cochetel *et al.*, the TCP20 homologue, which is RGM specific and not N-responsive, could be a key element for understanding RGM adaptation to N-poor soils. Very recently it was found that AtTCP20 interacts with NLP proteins and controls root development in N-deficient conditions (Guan *et al.*, 2017). If this TCP20 homologue in RGM determines its aptitude to low-N adaptation, responding very rapidly to high nitrate supply, it might open new possibilities for rootstock selection.

## An N-responsive, tri-partite system

Ruffel *et al.* (2011) identified sets of genes responding to the local or systemic response to nitrate availability, and the split-root system used by Cochetel *et al.* is an extremely useful tool for exploring this area. It is now clear that the root systemic response involves some molecular actors expressed in the foliar part of the plant (Ruffel *et al.*, 2011, 2016; Ohkubo *et al.*, 2017). The scion–rootstock combination effect arises from a bi-directional interaction, in which signals coming from the roots affect the scion physiology and vice versa. Adding the nitrate-heterogeneous compartments of the split root, we obtain a tri-partite system (the scion and the two parts of the split-root system). In a heterogeneous soil, a nutrient signal coming from one side of the root system should reach the distant parts of the root passing through the scion. Following this idea, it would be remarkable if the different responses to nitrate supply observed in CS/1103P and CS/RGM grafting combinations were due to a scion element that differentially interacts with the two rootstocks. Cochetel *et al.* point out the strong responsiveness of N-related genes (e.g. NRT2.4a, NPF6.3) under high nitrate treatment in RGM compared to 1103P. It is an open question as to whether this high N-responsiveness is explained by the fact that CS also integrates (in an additive way) the low nitrate information coming from the other side of the RGM roots, but not from the 1103P roots. However, completion of the transcriptomic data with CS/RGM and CS/1103P under split homogeneous conditions (high and low nitrate) will provide the answer.

## New directions: not just nitrate

High-throughput methodologies are powerful tools for understanding the complex response to nutrient availability. Thinking and focusing on a single response or pathway is too restrictive – the system is more complex and able to integrate different environmental signals from the roots, which often share points of cross-talk (Kellermeier *et al.*, 2014; Briat *et al.*, 2015). Interestingly, the GARP transcription factor function is among the gene categories enriched for 1103P and RGM rootstocks under heterogeneous N supply. Recently it was demonstrated that GARP transcription factors are at the convergence between nitrate and phosphate signals in Arabidopsis (Medici *et al.*, 2015; Nagarajan *et al.*, 2016). In addition, the authors found that strigolactone biosynthesis genes were among the most tightly regulated in low nitrate conditions, in a rootstock-dependent manner. Strigolactones are important hormonal regulators of the phosphate starvation response (Sun *et al.*, 2014; Kumar *et al.*, 2015). Since N and P are fundamental macronutrients for plant biomass determination, the findings of Cochetel *et al.* suggest that a fine tuning of the three steps of acquisition, assimilation and allocation of N and P mineral forms takes place in grapevine rootstocks and is linked to the control of the scion growth. The close relationship between N and P levels in grafted grapevines was already studied in a root ionome analysis on the same varieties, which showed that N and P are finely modulated and influenced by the rootstock (Lecourt *et al.*, 2015). Two particular unanswered questions remain. First, which molecular elements are responsible for the communication between rootstock and scion? And second, how is the N/P balance maintained even in a two-species assembly? Different mobile molecules such as hormones, miRNAs, proteins or small peptides are already targets of investigation as mobile elements connecting leaves and roots in non-grafted plants. These might also be good targets for understanding scion–rootstock communication. More specifically, SSPs (Small Secretory Peptides) and in particular CLE and CEP peptides, which are known to be regulated by multiple nutrient deficiencies and involved in the autoregulation of mycorrhization in different species (de Bang *et al.*, 2017), are emerging as important long-distance signalling molecules and will certainly receive increasing attention in this research area.

## References

[CIT0001] Araus V, Vidal EA, Puelma T, Alamos S, Mieulet D, Guiderdoni E, Gutiérrez RA 2016 Members of BTB gene family of scaffold proteins suppress nitrate uptake and nitrogen use efficiency. Plant Physiology 171, 1523–1532. 27208309 10.1104/pp.15.01731 PMC4902579

[CIT0002] Briat JF, Rouached H, Tissot N, Gaymard F, Dubos C 2015 Integration of P, S, Fe, and Zn nutrition signals in *Arabidopsis thaliana*: potential involvement of PHOSPHATE STARVATION RESPONSE 1 (PHR1). Frontiers in Plant Science 5, 290. 10.3389/fpls.2015.00290 PMC4411997 25972885

[CIT0003] Canales J, Moyano TC, Villarroel E, Gutiérrez RA 2014 Systems analysis of transcriptome data provides new hypotheses about *Arabidopsis* root response to nitrate treatments. Frontiers in Plant Science 5, 22. 24570678 10.3389/fpls.2014.00022 PMC3917222

[CIT0004] Cochetel N, Escudié F, Cookson SJ, Dai Z, Vivin P, Bert P-F, Muñoz MS, Delrot S, Klopp C, Ollat N, Lauvergeat V 2017 Root transcriptomic responses of grafted grapevines to heterogeneous nitrogen availability depend on rootstock genotype. Journal of Experimental Botany 68, 4339–4355. 28922755 10.1093/jxb/erx224 PMC5854021

[CIT0005] de Bang TC, Lay KS, Scheible W-R, Takahashi H 2017 Small peptide signaling pathways modulating macronutrient utilization in plants. Current Opinion in Plant Biology 39, 31–39. 28582679 10.1016/j.pbi.2017.05.005

[CIT0006] Guan P, Ripoll J-J, Wang R, Vuong L, Bailey-Steinitz LJ, Ye D, Crawford NM 2017 Interacting TCP and NLP transcription factors control plant responses to nitrate availability. Proceedings of the National Academy of Sciences, USA 114, 2419–2424. 10.1073/pnas.1615676114 PMC5338533 28202720

[CIT0007] Gutiérrez RA, Stokes TL, Thum K 2008 Systems approach identifies an organic nitrogen-responsive gene network that is regulated by the master clock control gene CCA1. Proceedings of the National Academy of Sciences, USA 105, 4939–4944. 10.1073/pnas.0800211105 PMC2290744 18344319

[CIT0008] Kellermeier F, Armengaud P, Seditas TJ, Danku J, Salt DE, Amtmann A 2014 Analysis of the root system architecture of Arabidopsis provides a quantitative readout of crosstalk between nutritional signals. The Plant Cell 26, 1480–1496. 24692421 10.1105/tpc.113.122101 PMC4036566

[CIT0009] Kumar M, Pandya-Kumar N, Kapulnik Y, Koltai H 2015 Strigolactone signaling in root development and phosphate starvation. Plant Signaling & Behavior 10, e1045174. 26251884 10.1080/15592324.2015.1045174 PMC4622057

[CIT0010] Lecourt J, Lauvergeat V, Ollat N, Vivin P, Cookson SJ 2015 Shoot and root ionome responses to nitrate supply in grafted grapevines are rootstock genotype dependent. Australian Journal of Grape and Wine Research 21, 311–318.

[CIT0011] Medici A, Marshall-Colon A, Ronzier E, Szponarski W, Wang R, Gojon A, Crawford NM, Ruffel S, Coruzzi GM, Krouk G 2015 AtNIGT1/HRS1 integrates nitrate and phosphate signals at the Arabidopsis root tip. Nature Communications 6, 6274. 10.1038/ncomms7274 PMC4373655 25723764

[CIT0012] Mudge K, Janick J, Scofield S, Goldschmidt EE 2009 A history of grafting. In: Janick J, ed. Horticultural Reviews, Vol. 35 437–493. John Wiley & Sons, Inc., Hoboken, NJ, USA. doi:10.1002/9780470593776.ch9.

[CIT0013] Nagarajan VK, Satheesh V, Poling MD, Raghothama KG, Jain A 2016 Arabidopsis MYB-related HHO2 exerts a regulatory influence on a subset of root traits and genes governing phosphate homeostasis. Plant and Cell Physiology 57, 1142–1152. 27016098 10.1093/pcp/pcw063

[CIT0014] O’Brien JA, Vega A, Bouguyon E, Krouk G, Gojon A, Coruzzi G, Gutiérrez RA 2016 Nitrate transport, sensing, and responses in plants. Molecular Plant 9, 837–856. 27212387 10.1016/j.molp.2016.05.004

[CIT0015] Ohkubo Y, Tanaka M, Tabata R, Ogawa-Ohnishi M, Matsubayashi Y 2017 Shoot-to-root mobile polypeptides involved in systemic regulation of nitrogen acquisition. Nature Plants 3, 17029. 28319056 10.1038/nplants.2017.29

[CIT0016] Ruffel S, Krouk G, Ristova D, Shasha D, Birnbaum KD, Coruzzi GM 2011 Nitrogen economics of root foraging: transitive closure of the nitrate–cytokinin relay and distinct systemic signaling for N supply vs. demand. Proceedings of the National Academy of Sciences, USA 108, 18524–18529. 10.1073/pnas.1108684108 PMC3215050 22025711

[CIT0017] Ruffel S, Poitout A, Krouk G, Coruzzi GM, Lacombe B 2016 Long-distance nitrate signaling displays cytokinin dependent and independent branches. Journal of Integrative Plant Biology 58, 226–229. 26619818 10.1111/jipb.12453

[CIT0018] Sun H, Tao J, Liu S, Huang S, Chen S, Xie X, Yoneyama K, Zhang Y, Xu G 2014 Strigolactones are involved in phosphate- and nitrate-deficiency-induced root development and auxin transport in rice. Journal of Experimental Botany 65, 6735–6746. 24596173 10.1093/jxb/eru029 PMC4246174

[CIT0019] Warschefsky EJ, Klein LL, Frank MH, Chitwood DH, Londo JP, von Wettberg EJB, Miller AJ 2016 Rootstocks: diversity, domestication, and impacts on shoot phenotypes. Trends in Plant Science 21, 418–437. 26698413 10.1016/j.tplants.2015.11.008

[CIT0020] Zhang X, Davidson EA, Mauzerall DL, Searchinger TD, Dumas P, Shen Y 2015 Managing nitrogen for sustainable development. Nature 528, 51–59. 26595273 10.1038/nature15743

